# A Rare Case of Fluoxetine-Induced Vortex Keratopathy

**DOI:** 10.7759/cureus.102975

**Published:** 2026-02-04

**Authors:** Soumya Behera, Subhangi Sahu, Somya Pilani, Apoorva V

**Affiliations:** 1 Ophthalmology, Kalinga Institute of Medical Sciences, Bhubaneswar, IND

**Keywords:** adverse effects, cornea, cornea verticillata, drug effects, fluoxetine, ssri, vortex keratopathy

## Abstract

Vortex keratopathy is a distinctive corneal epithelial disorder marked by whorl-shaped deposits, most often linked to prolonged exposure to cationic amphiphilic medications. While classic drug associations are well established, the involvement of selective serotonin reuptake inhibitors remains sparsely documented. This report describes an unusual presentation of vortex keratopathy temporally associated with long-term fluoxetine 40 mg therapy for two years.

A 25-year-old woman receiving treatment for depressive disorder presented with gradually progressive bilateral visual disturbance. Comprehensive ocular examination revealed preserved best-corrected visual acuity but demonstrated bilateral, symmetrical, and vortex-patterned epithelial corneal deposits. Anterior segment optical coherence tomography localized hyperreflective changes to the corneal epithelium, with deeper layers remaining unaffected. No systemic disease, hereditary corneal disorder, or alternative medication known to cause cornea verticillata was identified. The patient had been continuously using oral fluoxetine for two years.

After coordinated discussion with psychiatric services, fluoxetine was withdrawn and replaced with an alternative antidepressant. Conservative ophthalmic management with preservative-free lubricants was initiated, alongside patient counseling regarding prognosis and reversibility. Subsequent follow-up demonstrated progressive symptomatic improvement and partial regression of epithelial deposits on slit-lamp examination, without compromise of visual acuity or emergence of additional ocular pathology.

This case underscores the importance of meticulous drug history assessment in unexplained corneal epithelial disorders. Although rare, fluoxetine-associated vortex keratopathy should be recognized as a reversible adverse effect, enabling timely intervention and coordinated multidisciplinary care.

## Introduction

Corneal deposits constitute important ocular conditions associated with vision deficits, the most common of which is vortex keratopathy. Vortex keratopathy, also known as cornea verticillata, primarily affects the corneal epithelium and involves whorl-like deposits on its surface. These golden-brown or gray opacities are often asymptomatic but may also present as progressive blurring of vision. The clinical pattern can be explained by the centripetal migration of deposit-laden limbal stem cells as the corneal epithelium undergoes natural growth and repair [1].

Vortex keratopathy is known to be associated with certain medications, metabolic substrates, or disease byproducts in the basal epithelial layer of the cornea [2]. However, the most common associations are pharmacological. Amiodarone, hydroxychloroquine, chloroquine, indomethacin, and phenothiazines are commonly implicated [1]. Topical rho-kinase inhibitors are also known to cause cornea verticillata [3]. Other less commonly associated agents include gentamicin, tamoxifen, meperidine, chlorpromazine, atovaquone, suramin, tilorone, perhexiline maleate, tyrosine kinase inhibitors, vandetanib, osimertinib, and, very rarely, SSRIs (selective serotonin reuptake inhibitors) like fluoxetine [4-12].

The medications that produce cornea verticillata share cationic, amphiphilic properties that allow them to penetrate lysosomes in the basal epithelial layer of the cornea, where they bind to cellular lipids. These medication-lipid complexes are resistant to enzymatic degradation and accumulate as deposits in the cornea. Lipophilic properties of amiodarone [4,5], chloroquine, chlorpromazine, quinacrine, suramin, and atovaquone are implicated in the etiology of vortex keratopathy associated with these drugs [6]. A case of keratopathy associated with ribociclib was reported in a patient with bony metastasis of breast cancer. However, the probable pathogenic mechanisms were not discussed [7]. Vandetanib has also been implicated in a case of keratopathy in another case report, where pathogenic pathways, such as the effect of anti-EGFR (estimated glomerular filtration rate) mechanisms in corneal epithelial structure and physiology, have been discussed [8]. Similarly, case reports for novel anticancer drugs [9], raloxifene [10], netarsudil [11], and hydroxychloroquine [12] as etiological factors in vortex keratopathy exist.

Non-pharmacological etiologies include multiple myeloma, multiple sulfatase deficiency [13], generalized gangliosidosis (Fabry disease), neurotrophic keratitis, Lisch corneal dystrophy, epidemic keratoconjunctivitis, and iron and stromal depositions (gold, silver, antacid, and retinoid depositions). Fabry keratopathy is the most common ophthalmic manifestation in Fabry disease and shows progression of ocular defect over the years [14,15]. On the other hand, cases have been reported in association with multiple myeloma [16,17].

Despite its association with medication, cases of vortex keratopathy related to SSRIs are rare. Antidepressant drugs, including SSRIs and fluoxetine, have been implicated sporadically in studies [18-20]. In this study, we report a case of vortex keratopathy associated with fluoxetine.

## Case presentation

A 25-year-old female with a documented history of depressive disorder was referred from the psychiatry outpatient department of a teaching hospital in eastern India to the ophthalmology outpatient department with a primary complaint of progressive blurring of vision in both eyes. The patient reported that the visual disturbance had developed insidiously over time and was not associated with acute onset, pain, redness, photophobia, watering, foreign body sensation, or diplopia. There was no history of trauma, ocular surgery, contact lens use, or exposure to chemical or occupational hazards. She denied any systemic illnesses such as diabetes mellitus, hypertension, thyroid disorders, or autoimmune conditions. There was no relevant family history of corneal dystrophies or degenerative ocular diseases.

On ophthalmic examination, her best-corrected visual acuity was recorded as 6/6 for distance and N6 for near vision in both eyes, indicating preservation of central visual acuity despite her subjective visual complaints. Pupillary reactions were normal and symmetrical, and extraocular movements were full in all gazes. Intraocular pressure, measured using applanation tonometry, was within normal physiological limits in both eyes. Tear film evaluation did not reveal any significant signs of dry eye disease.

**Figure 1:**
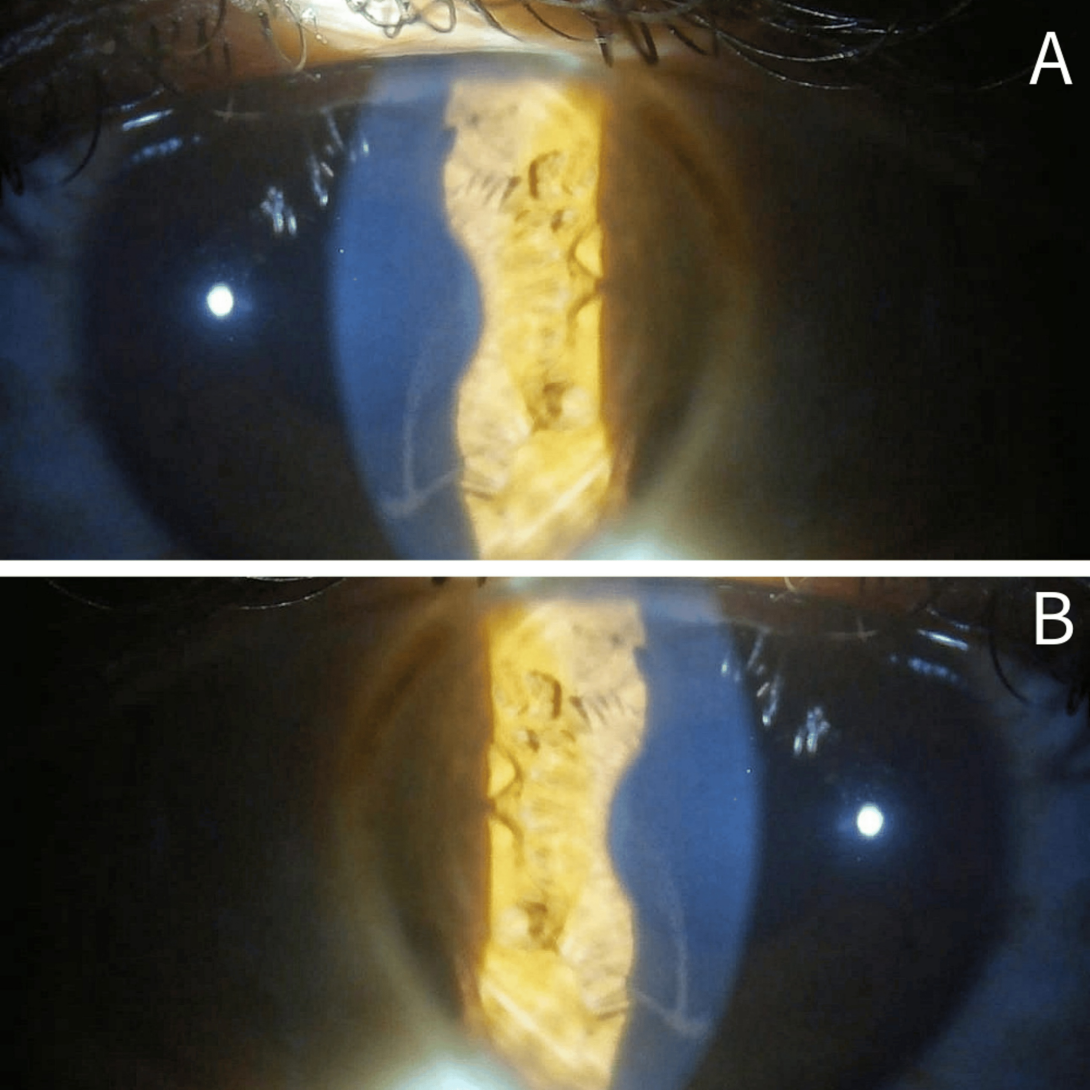
Slit-lamp biomicroscopy (diffuse illumination) of the right (A) and left (B) eyes demonstrating bilateral, golden-brown, curvilinear epithelial corneal deposits arranged in a characteristic whorl-like (vortex) pattern radiating centrifugally from the central cornea, consistent with cornea verticillata. The corneal epithelium appears intact, with clear underlying stroma, no associated corneal edema, and a quiet anterior chamber.

Slit-lamp biomicroscopic examination revealed a distinctive pattern of whorl-like, grayish-white epithelial deposits arranged in a vortex configuration over the central and paracentral cornea of both eyes. These corneal changes were confined to reveal a characteristic appearance consistent with vortex keratopathy. The corneal stroma appeared clear, with no evidence of edema, scarring, or infiltrates. The endothelium was unremarkable on specular reflection. Examination of the conjunctiva, sclera, iris, and lens showed no abnormalities. Posterior segment evaluation with dilated fundus examination demonstrated a healthy optic disc, normal retinal vasculature, and an intact macula in both eyes (Figure 1).

**Figure 2:**
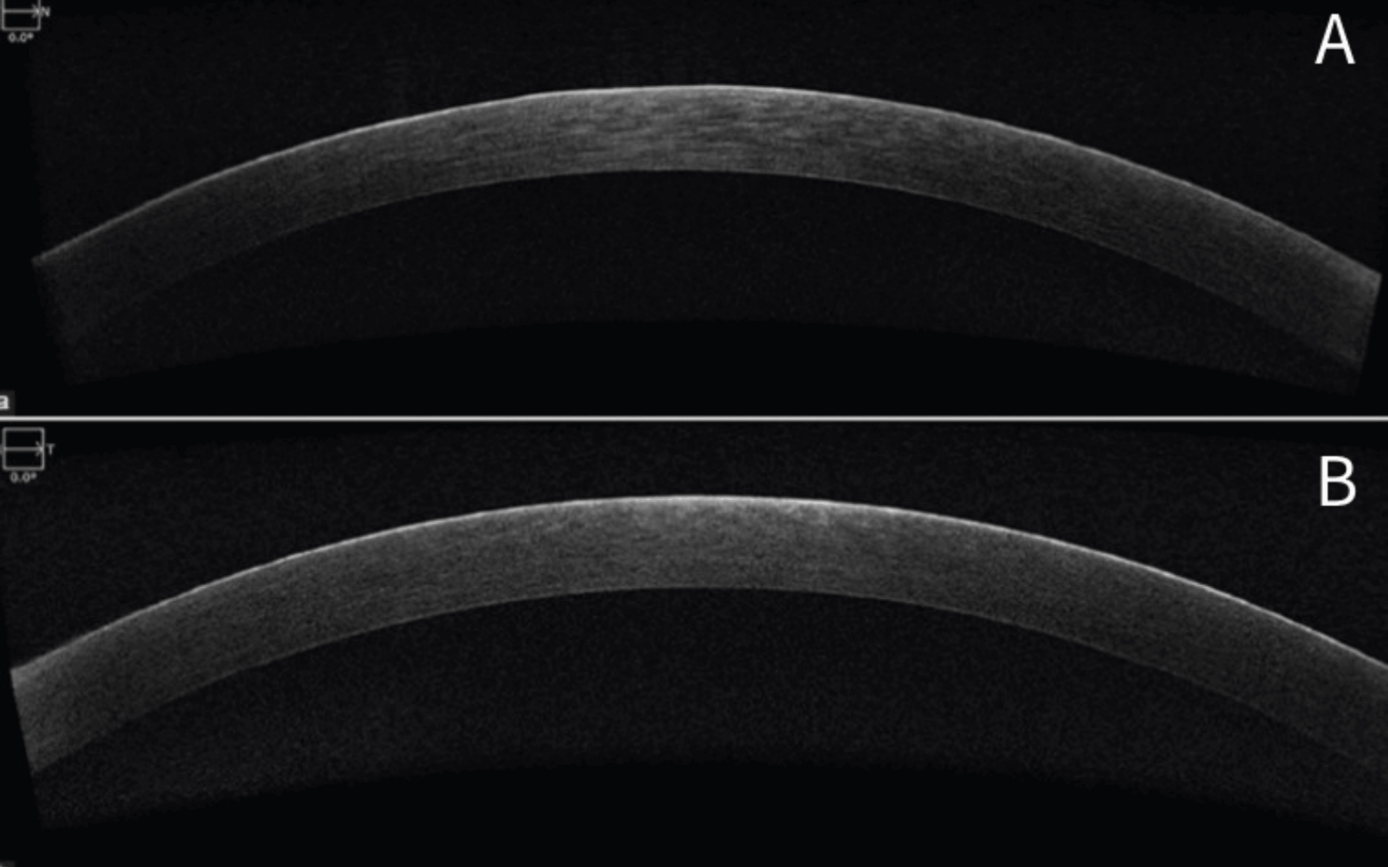
AS-OCT images of the right (A) and left (B) eyes demonstrating bilateral hyperreflective band-like deposits within the corneal epithelium This image shows the anterior segment optical coherence tomography (AS-OCT) of the right and left eyes demonstrating hyperreflective bands confined to the corneal epithelial layer, with preservation of Bowman’s layer and underlying stroma.

To further characterize the corneal findings, anterior segment optical coherence tomography (AS-OCT) was performed. Imaging revealed well-defined hyperreflective bands localized to the corneal epithelial layer bilaterally, with preservation of the underlying Bowman’s layer and stroma (Figure 2). These findings supported the slit-lamp impression of epithelial deposition rather than deeper corneal pathology. Corneal sensation was intact, and no signs suggestive of infectious or inflammatory keratitis were present.

A detailed review of the patient’s medication history revealed long-term pharmacological treatment for depressive disorder. She had been taking the tablet fluoxetine at a dose of 40 mg once daily continuously for the past two years. There was no history of use of other systemic medications classically associated with vortex keratopathy, such as amiodarone, chloroquine, hydroxychloroquine, tamoxifen, or indomethacin. Additionally, she was not on any topical ocular medications. Based on the temporal association between prolonged fluoxetine therapy and the onset of corneal findings, a drug-induced etiology was strongly suspected.

Fluoxetine is an SSRI that is widely prescribed for depressive and anxiety disorders. While it is generally considered safe, rare ocular adverse effects have been reported, predominantly involving accommodation disturbances, dry eye symptoms, and, very infrequently, corneal epithelial changes.

In consultation with the treating psychiatrist, a decision was made to discontinue fluoxetine, considering the suspected adverse ocular effect and the availability of alternative antidepressant options. The patient was counseled extensively regarding the benign nature of the corneal deposits, the expected reversibility of the condition, and the importance of psychiatric follow-up to ensure continuity of mental health care. Supportive ophthalmic management was initiated with preservative-free lubricating eye drops to improve ocular surface comfort and promote epithelial health.

**Figure 3:**
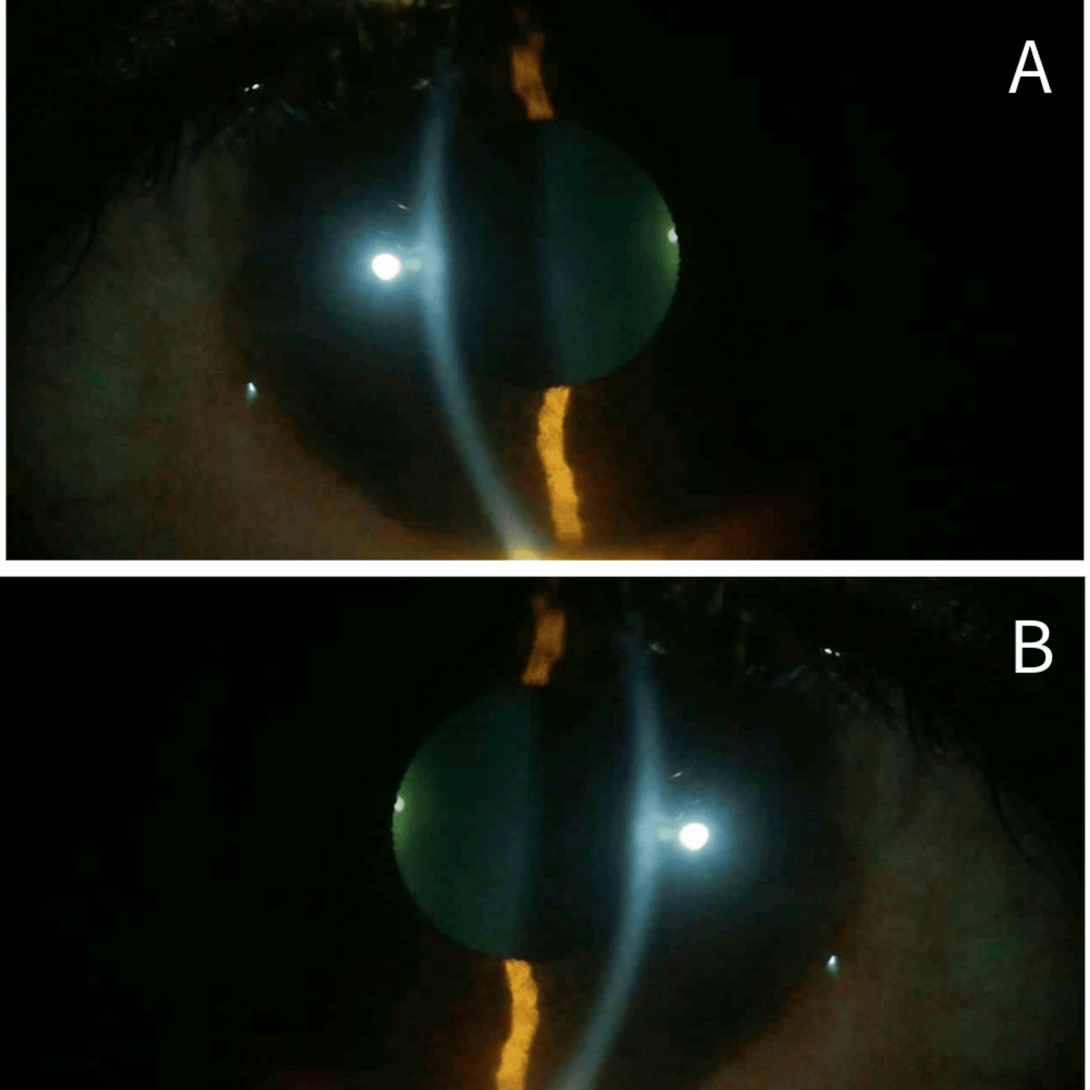
Slit-lamp biomicroscopy of the right (A) and left (B) eyes at three-month follow-up, demonstrating resolution of epithelial corneal deposits with restoration of a clinically clear cornea

On follow-up visits, the patient reported gradual subjective improvement in visual clarity. Repeat slit-lamp examination after three months demonstrated partial regression of the whorl-like epithelial deposits over time, consistent with the known reversibility of vortex keratopathy following withdrawal of the offending agent. No deterioration in visual acuity or emergence of new ocular signs was noted during the observation period (Figure 3).

## Discussion

Role of SSRIs in ocular defects

SSRIs are associated with various ocular defects. These antidepressants were associated with severe eye defects in around 38% of users. The highest incidence was reported in patients who were administered fluoxetine [19]. Research suggests a mandatory ophthalmologic consult for patients on SSRIs, especially in older individuals and persons with diagnosed glaucoma, due to the risk of changes in intraocular pressure [21]. However, another study by Karaküçük et al. was not able to confirm significant long-term effects of SSRIs on cornea and lens density, even though early effects were present [22]. SSRIs have also been associated with increased aggravation of dry eye [23]. Additionally, a study conducted in 18 countries showed that around 40% of the participants reported that the ocular effects caused an interference with their work [24]. SSRIs have also been reported to cause optic neuropathy [25].

Fluoxetine itself has been indicated in the etiology of many eye diseases in animal studies, as well as in-vitro and in-human studies. Fluoxetine increases intraocular pressure in rabbits [26]. Another case report has associated it with subhyaloid damage [27], while yet another report showed its association with maculopathy [28].

Pathophysiology of fluoxetine-induced vortex keratopathy

The pathogenesis of drug-induced vortex keratopathy is thought to involve intracellular accumulation of drug-lipid complexes within basal epithelial cells, leading to the characteristic whorl-like pattern observed clinically. The bilateral and symmetrical nature of the findings in this patient further supported a systemic drug-related cause. Fluoxetine is a cationic amphiphilic agent that can penetrate the cornea epithelium and bind to cellular lipids. The drug-lipid complex deposit accumulates within the lysosomes of basal epithelial cells of the cornea, forming whorl-like opacities resulting in cornea verticillata.

Importance of fluoxetine-induced vortex keratopathy

This case highlights the importance of a thorough drug history in patients presenting with unexplained corneal epithelial changes. Although fluoxetine-induced vortex keratopathy is exceedingly rare, awareness of this potential association is essential for early recognition and appropriate management. Fluoxetine is a SSRI. The common ocular side effects associated with fluoxetine are dry eye, blurring of vision, and mydriasis [29] due to 5HT1a receptor stimulation in the ciliary muscle, with a potential risk of ​​​​​angle-closure glaucoma. Fluoxetine increases central serotonin levels, which can shift autonomic balance toward relative sympathetic dominance and reduced parasympathetic activity. This may promote α₁-adrenergic-mediated contraction of the iris dilator muscle, leading to mydriasis and, in susceptible individuals, a risk of angle-closure glaucoma. Importantly, fluoxetine does not produce this effect through anticholinergic mechanisms. Maculopathy has also been reported with fluoxetine.

This study is limited by its single-case design and the absence of a formal causality assessment tool, which restricts the ability to establish a definitive drug-event relationship. Future reports would benefit from incorporating a structured causality assessment tool, such as the Naranjo Scale, to strengthen the objectivity of drug-event associations.

Timely identification and cessation of the causative medication can lead to resolution of corneal changes and prevent unnecessary investigations or interventions. Furthermore, interdisciplinary collaboration between ophthalmologists and psychiatrists plays a crucial role in balancing ocular safety with effective management of underlying psychiatric conditions.

## Conclusions

Fluoxetine-induced vortex keratopathy is an exceptionally rare but clinically important adverse ocular effect. This case highlights that long-term fluoxetine therapy can lead to characteristic bilateral corneal epithelial deposits, resulting in visual symptoms despite preserved visual acuity. Recognition of this entity relies on careful clinical examination supported by anterior segment imaging and, critically, a detailed drug history.

The condition is benign and potentially reversible, with gradual regression of corneal changes following timely discontinuation of the offending medication. Awareness of this rare association is essential for ophthalmologists and psychiatrists alike, as early diagnosis and interdisciplinary management can prevent unnecessary investigations, ensure visual recovery, and allow safe continuation of appropriate psychiatric care using alternative therapies.
